# Synthesis, antimicrobial activity, pharmacophore modeling and molecular docking studies of new pyrazole-dimedone hybrid architectures

**DOI:** 10.1186/s13065-018-0399-0

**Published:** 2018-03-14

**Authors:** Assem Barakat, Abdullah M. Al-Majid, Bander M. Al-Qahtany, M. Ali, Mohamed Teleb, Mohamed H. Al-Agamy, Sehrish Naz, Zaheer Ul-Haq

**Affiliations:** 1 Department of Chemistry, Faculty of Science, King Saud University, P. O. Box 2455, Riyadh, 11451 Saudi Arabia; 2 Department of Chemistry, Faculty of Science, Alexandria University, P. O. Box 426, Ibrahimia, 21321 Alexandria, Egypt; 3 Department of Pharmaceutical Chemistry, Faculty of Pharmacy, Alexandria University, Alexandria, 21521 Egypt; 4 Microbiology and Immunology Department, Faculty of Pharmacy, Al-Azhar University, Cairo, Egypt; 5 Division of Microbiology, Pharmaceutics Department, College of Pharmacy, King Saud University, P. O. Box 2457, Riyadh, 11451 Saudi Arabia; 6 Dr. Panjwani Center for Molecular Medicine and Drug Research, International Center for Chemical and Biological Sciences, University of Karachi, Karachi, 75210 Pakistan

**Keywords:** Pyrazole, Dimedone, Antifungal activity, Antimicrobial activity, Structure activity relationship, Inhibition mechanism prediction

## Abstract

**Background:**

Design and synthesis of pyrazole-dimedone derivatives were described by one-pot multicomponent reaction as new antimicrobial agents. These new molecular framework were synthesized in high yields with a broad substrate scope under benign conditions mediated by diethylamine (NHEt_2_). The molecular structures of the synthesized compounds were assigned based on different spectroscopic techniques (^1^H-NMR, ^13^C-NMR, IR, MS, and CHN).

**Results:**

The synthesized compounds were evaluated for their antibacterial and antifungal activities against *S. aureus* ATCC 29213, *E. faecalis* ATCC29212*, B. subtilis* ATCC 10400, and *C. albicans* ATCC 2091 using agar Cup plate method. Compound **4b** exhibited the best activity against *B. subtilis and E. faecalis* with MIC = 16 µg/L. Compounds **4e** and **4l** exhibited the best activity against *S. aureus* with MIC = 16 µg/L. Compound **4k** exhibited the best activity against *B. subtilis* with MIC = 8 µg/L. Compounds **4o** was the most active compounds against *C. albicans* with MIC = 4 µg/L.

**Conclusion:**

*In*-*silico* predictions were utilized to investigate the structure activity relationship of all the newly synthesized antimicrobial compounds. In this regard, a ligand-based pharmacophore model was developed highlighting the key features required for general antimicrobial activity. While the molecular docking was carried out to predict the most probable inhibition and binding mechanisms of these antibacterial and antifungal agents using the MOE docking suite against few reported target proteins.
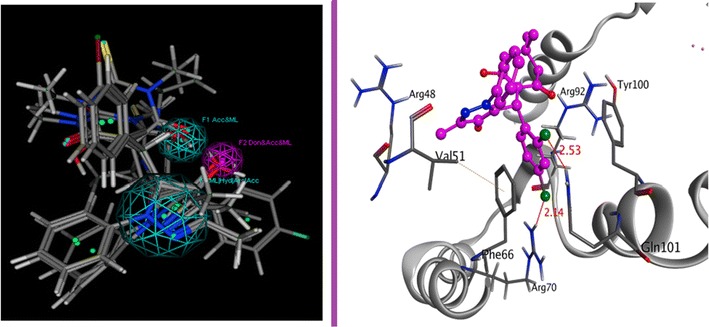

**Electronic supplementary material:**

The online version of this article (10.1186/s13065-018-0399-0) contains supplementary material, which is available to authorized users.

## Background

Nosocomial infections caused by antibiotic-resistant gram-positive bacteria have become a serious medical problem with an alarming increasing rate worldwide. Methicillin-resistant *Staphylococcus aureus* (MRSA), vancomycin-resistant enterococci (VRE) and penicillin-resistant *Streptococcus pneumoniae* (PRSP) are of particular concern among various hospital-acquired infections [1]. Accordingly, emerging investigations have provided new insights into developing novel, safe and effective antibacterial agents. Within this scope, pyrazole based antibacterial agents attracted great interest [2]. Generally, pyrazoles display innumerable pharmacological activities ranging from analgesic, antipyretic, antimicrobial, anti-inflammatory, anticancer effects to antidepressant, anticonvulsant, and selective enzyme inhibitory activities [2–11]. Recently, Barakat et al, have been reported novel pyrazole hybrid architectures as efficient antibacterial agents. Various pharmacophores were linked to the pyrazole core to build bioactive scaffolds [12, 13]. Within this approach, cyclic dicarbonyl compounds of the type dimedone have attracted our interest. Dimedone has been utilized successfully as pharmacophoric building block in various antimicrobial agents such as xanthenes [14, 15], substituted chromenes [16], macrocyclic metal complexes [17], quinazoline derivatives [18], tetrahydro quinolone diones [19] and acridine based compounds [20]. Recognizing these facts and in continuation of our previous work [12, 13] new hybrid molecules incorporating pyrazoles and dimedone in a single molecular framework were designed and synthesized. We subjected our target compounds to pharmacophore modeling and molecular docking on different target proteins to explore their mode of action.

## Results and discussion

### Chemistry

**Scheme 1:**
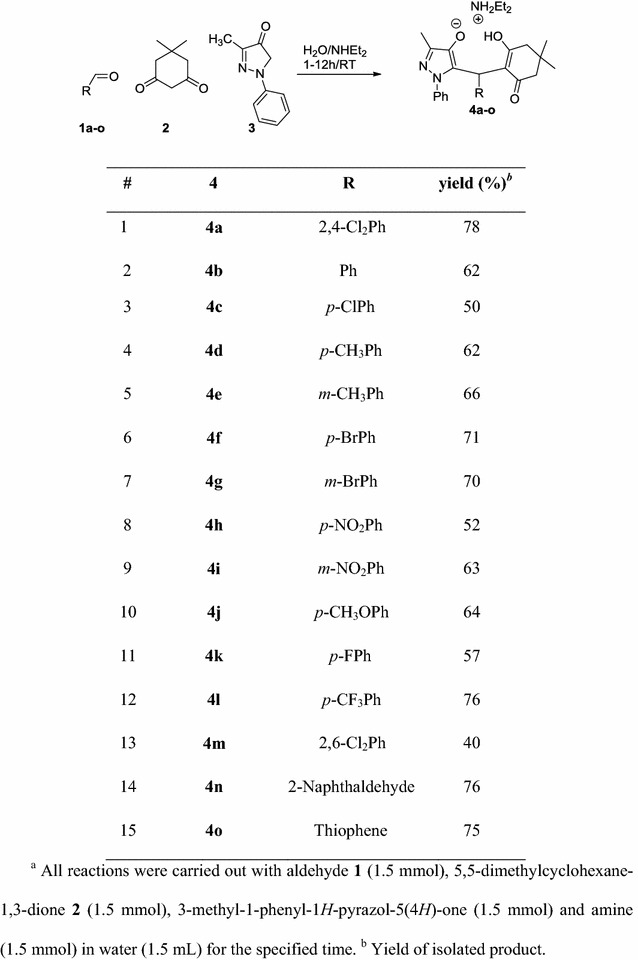
Substrate scope of the cascade reaction: variation of pyrazole-dimedone adducts

The designed bioactive scaffolds were synthesized utilizing green approach. The pyrazole-dimedone derivatives were prepared as shown in Scheme 1 via one pot Knoevenagel condensation Michael addition of 3-methyl-1-phenyl-1*H*-pyrazol-5(4*H*)-one, 1,3-dicarbonyl compound (dimedone) and various aldehydes mediated by aqueous NHEt_2_. This one pot multicomponent reaction afforded the final targets as hybrid frameworks **4a–o** in good yields (40–78%) with substrate tolerance of pyrazole-dimedone derivatives. The chemical structures of all the synthesized compounds were assigned by the aid of physical and spectroscopic methods (^1^H-NMR, ^13^C-NMR, IR, and elemental analyses).

**Scheme 2:**
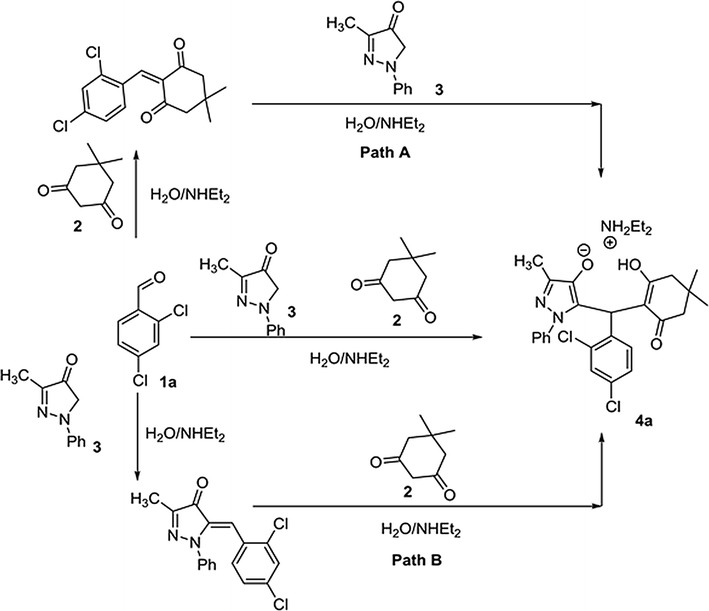
Possible mechanisms for the tandem Aldol-Michael reaction

The suggested mechanisms for obtaining the target compounds are shown in Scheme 2. Olefin is formed by Knoevenagel condensation of aryl aldehyde **1** and 1,3-diketone **2** to give benzylidenecyclohexandione intermediate which acts as a Michael acceptor. This Michael acceptor is attached by 3-methyl-1-phenyl-1*H*-pyrazol-5(4*H*)-one **3** (Michael donor) to give the requisite final targets **4a** (Path A). Another bath way is Knoevenagel condensation between aryl aldehyde **1** and 3-methyl-1-phenyl-1*H*-pyrazol-5(4*H*)-one **3** to generate benzylidenepyrazolone intermediate which acts as a Michael acceptor. This Michael acceptor is attacked by 1,3-diketone **2** (Michael donor) to afford the final product **4a** (Path B).

### Antimicrobial activity

The synthesized pyrazole-dimedone derivatives showed various antibacterial activities. Results of the bactericidal activity are shown in Table 1; the minimum inhibitory concentration (MIC) results are expressed as µg/L inhibition.

**Table 1:** Results of cup-plate method expressed as minimum inhibitory concentrations (MIC) of the compounds in (μg/L)

Entry	Compounds	Gram positive bacteria						Yeast	
		*S. aureus* ATCC 29213		*E. faecalis* ATCC29212		*B. subtilis* ATCC10400		*C. albicans* ATCC 2091	
		CPM (mm)	MIC (µg/L)	CPM (mm)	MIC (µg/L)	CPM (mm)	MIC (µg/L)	CPM (mm)	MIC (µg/L)
1	*4a*	13	32	14	32	12	32	14	32
2	*4b*	15	32	13	16	15	16	15	32
3	*4c*	13	32	24	32	16	32	15	16
4	*4d*	16	32	16	32	18	32	16	16
5	*4e*	19	16	15	32	14	64	14	32
6	*4f*	14	32	13	64	15	32	14	32
7	*4g*	14	32	15	32	16	32	14	32
8	*4h*	12	64	14	32	16	32	17	16
9	*4i*	14	32	12	64	17	32	14	32
10	*4j*	10	64	13	32	10	32	13	32
11	*4k*	13	32	13	32	20	8	15	16
12	*4l*	16	16	16	32	16	32	14	32
13	*4m*	15	32	13	32	12	32	16	16
14	*4n*	14	32	13	32	15	32	14	32
15	*4o*	13	32	20	32	15	16	21	4
STD	Ciprofloxacin	27	≤ 0. 25	24	≤ 0.25	25	≤ 0.25	ND	ND
	Fluconazole	ND	ND	ND	ND	ND	ND	28	0.5

### Antibacterial activity against gram positive bacteria

The antibacterial activity of the novel pyrazole-dimedone compounds were evaluated against gram positive bacteria including *E. faecalis* ATCC29212*, S. aureus* ATCC 29213, and *B. subtilis* ATCC 10400. Ciprofloxacin was used as standard drug.

The results listed in Table 1 revealed that all pyrazole-dimedone compounds were active against the tested-strains including *S. aureus*, *E. faecalis*, and *B. subtilis*. Pyrazole-dimedone **4k** was the most active compound against *B. subtilis* with MIC value of 8 µg/L. Compounds **4e** and **4l** having 3-methyl and 4-trifluromethyl substituents on the phenyl ring respectively exhibited good activity against *S. aureus* with MIC value of 16 µg/L. Compounds **4a-d, 4f,g,i,k** and **4m–o** showed relatively lower activity against *S. aureus* with MIC value of 32 µg/L. Compounds **4h** and **4j** having 4-nitro and 4-methoxy substituents on the phenyl ring were the least active derivatives against *S. aureus* with MIC values of 64 µg/L. Compound **4b** bearing unsubstituted phenyl ring exhibited good activity against *E. faecalis* with MIC values of 16 µg/L. Compounds **4a, c–e, 4g, h** and **4j–o** showed lower activity against *E. faecalis* with MIC value of 32 µg/L. Compounds **4f** and **4i** having 4-bromo and 3-nitro substituents on the phenyl ring respectively were shown as the least active derivatives against *E. faecalis* with MIC value of 64 µg/L.

Substituted pyrazole-dimedone **4b** without substituent on the phenyl ring and **4o** having thiophene ring exhibited good activity against *B. subtilis* with MIC value of 16 µg/L. Compounds **4a, c, d, 4f–j** and **4l–o** showed lower activity against *B. subtilis* with MIC value of 32 µg/L. Compound **4e** having 3-methyl substituent on the phenyl ring was shown to be the least active against *B. subtilis* with MIC value of 64 µg/L.

### Antifungal activity

The newly synthesized pyrazole-dimedone derivatives were evaluated for their antifungal activity against fungi *C. albicans* (ATCC 2091) by the diffusion agar and serial dilution method (BSAC, 2015) [23] Fluconazole was used as standard antifungal agent. Results shown in Table 1 revealed that all pyrazole-dimedone compounds **4a-o** were active against the tested-strains *C. albicans* ATCC 2091. Pyrazole-dimedone **4o** bearing thiophene was the most active compounds from this series against *C. albicans* ATCC 2091 with MIC value of 4 µg/L. Compounds **4c, d, h, k, m** possessed good activity against *C. albicans* with MIC values of 16 µg/L. Compounds **4a, b, 4e–g,** and **4i, j, g, n** were the least active among this series as antifungal agent with MIC values of 32 µg/L.

### Structure activity relationship profiling via pharmacophore modeling

**Fig. 1:**
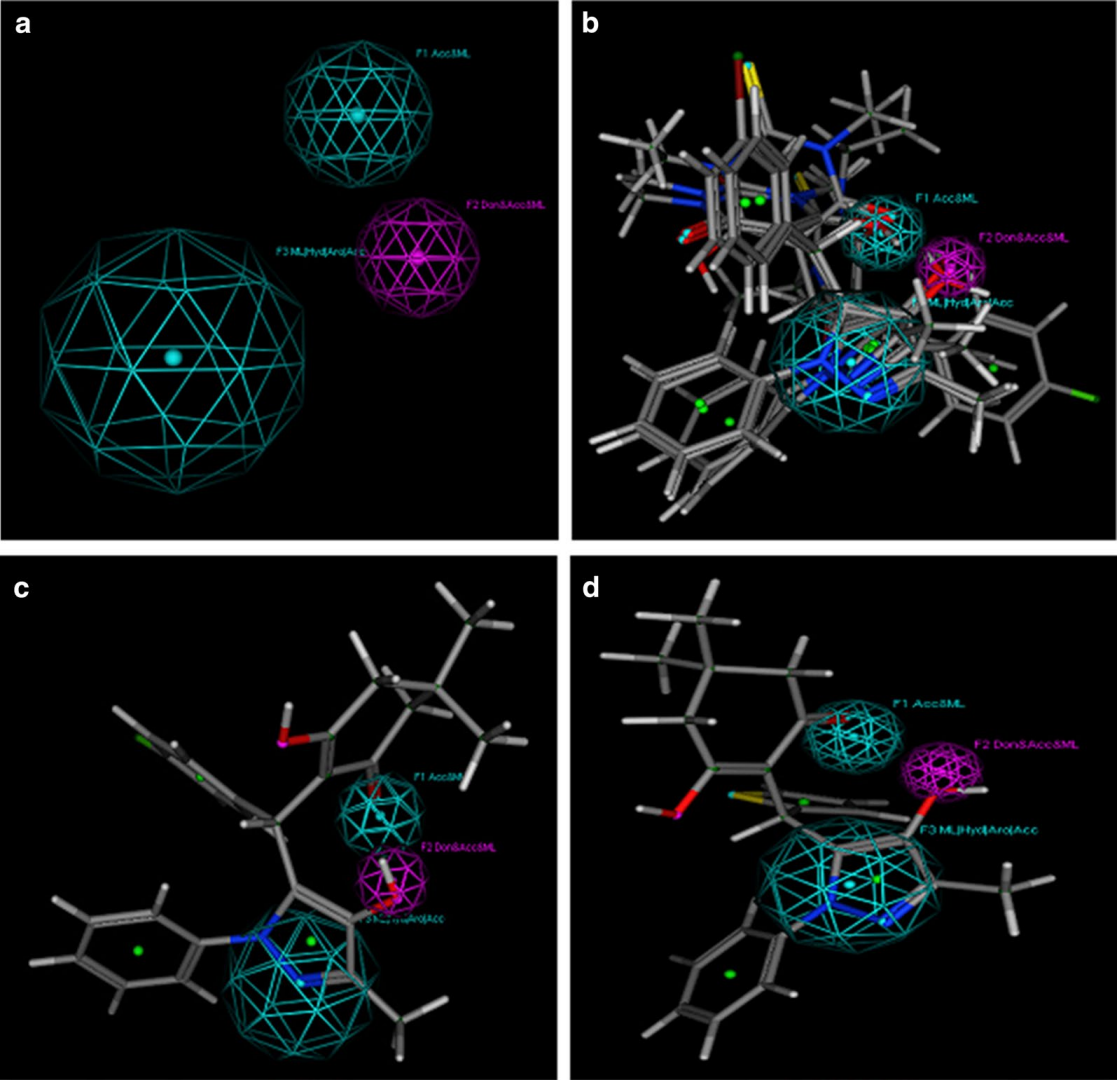
**a** Best query displaying pharmacophoric features shared by active lead compounds as colored spheres (cyan for hydrogen bond acceptor function with metal ligator (F1: Acc& ML), pink for hydrogen bond acceptor/donor function with metal ligator (F2: Don, Acc& ML) as well as cyan for hydrophobic region with aromatic centre, hydrogen bond acceptor or metal ligator function (F3: ML/Hyd/Aro/Acc). **b** Validation of the selected query; mapping of previously reported active compounds **4a** and **4n** [12] as well as **4a** and **4f** [13], showing RMSD values in acceptable range (0.2823-0.4993). **c** Mapping of compound **4k** on pharmacophore model. **d** Mapping of compound **4o** on pharmacophore model

First of all, to predict the structure activity relationship (SAR) of all the newly synthesized antimicrobial compounds, a ligand-based pharmacophore model was developed. This is the most reliable way to design new potent active molecules having similar scaffolds by utilizing their biological data in computational predictions. In this study, the selected pharmacophore including one hydrogen bond acceptor (F1: Acc& ML), one hydrogen bond donor (F2: Don, Acc& ML) and one hydrophobic feature with an aromatic center (F3: ML/Hyd/Aro) (Fig. 1a) was mapped over active compounds (Fig. 1b). The mapping was evaluated on the basis of their lowest RMSD between query and matching annotations (Fig. 1c, d).

The lowest RMSD indicates better compound fitness to the selected model. Results in Table 2 showed that all the active compounds were able to satisfy the pharmacophoric features of the generated model with RMSD values ranging from 0.3907 to 0.6571 Å along with their most suitable alignment of each compound over query. These results indicated the critical role of aromatic ring substitution which greatly effects the spatial orientation of cyclohexane ring with respect to the pyrazole moiety. This might be the best explanation to understand the differences in their respective antimicrobial activity profile.

**Table 2:** RMSD values along with their suitable alignment for Hit Compounds

Comp. no.	**4b**	**4c**	**4d**	**4e**	**4h**	**4k**	**4l**	**4m**	**4o**
RMSD (Å)	*0.3907*	*0.4715*	*0.4639*	*0.4663*	*0.4662*	*0.5938*	*0.5070*	*0.6571*	*0.5660*

### Docking simulation to predict the mode of inhibition

After SAR profiling, docking studies were carried out to predict the most suitable binding pose and inhibition mechanism of newly synthesized derivatives. But before docking, based on the principle that similar Compounds tend to bind to the same proteins, we predicted few protein targets reported against reference compounds (ciprofloxacin and fluconazole) and docked our active compounds against them. Binding DB brought in seven different target proteins i.e. Dihydrofolate Reductase (DHFR) (PDB ID 4HOF), Secreted Aspartic Protease (PDB ID 3Q70), and *N*-myristoyl Transferase (PDB ID 1IYL) from *C. Albicans* as fungal target together with Dihydrofolate Reductase (PDB ID 3FYV), Gyrase B (PDB ID 4URM), Thymidylate Kinase (TMK) (PDB ID 4QGG) and Sortase A (PDB ID 2MLM) from *S. aureus* as bacterial target. Among all these seven proteins, only two proteins i.e. one proteins (Thymidylate Kinase) from *S. aureus* [21] and one protein (*N*-myristoyl transferase) from *C. albican* [22] presented good binding affinity, while all other targets showed very few or no interactions with these derivatives.

The potencies of these newly synthesised derivatives were measured computationally in terms of their dock Scores. Dock score which is actually the strength of the non-covalent interactions among multiple molecules within the binding pocket of a target protein. The more negative the score is, the more favorable interactions between compound and the target protein are. Here in our study, the compound **4l** being the most potent antibacterial agent against TMK (ID: 4QGG) from *S. aurues*, displayed the highest negative score of − 6.86 kcal/mol which is comparable of the standard drug ciprofloxacin with the score of − 6.9 kcal/mol. Similarly, **4o** being the most potent antifungal agent displayed good docking score of − 8.7 kcal/mol and molecular interactions with *N*-myristoyl transferase (NMT) enzyme from *C. Albicans.*

**Fig. 2:**
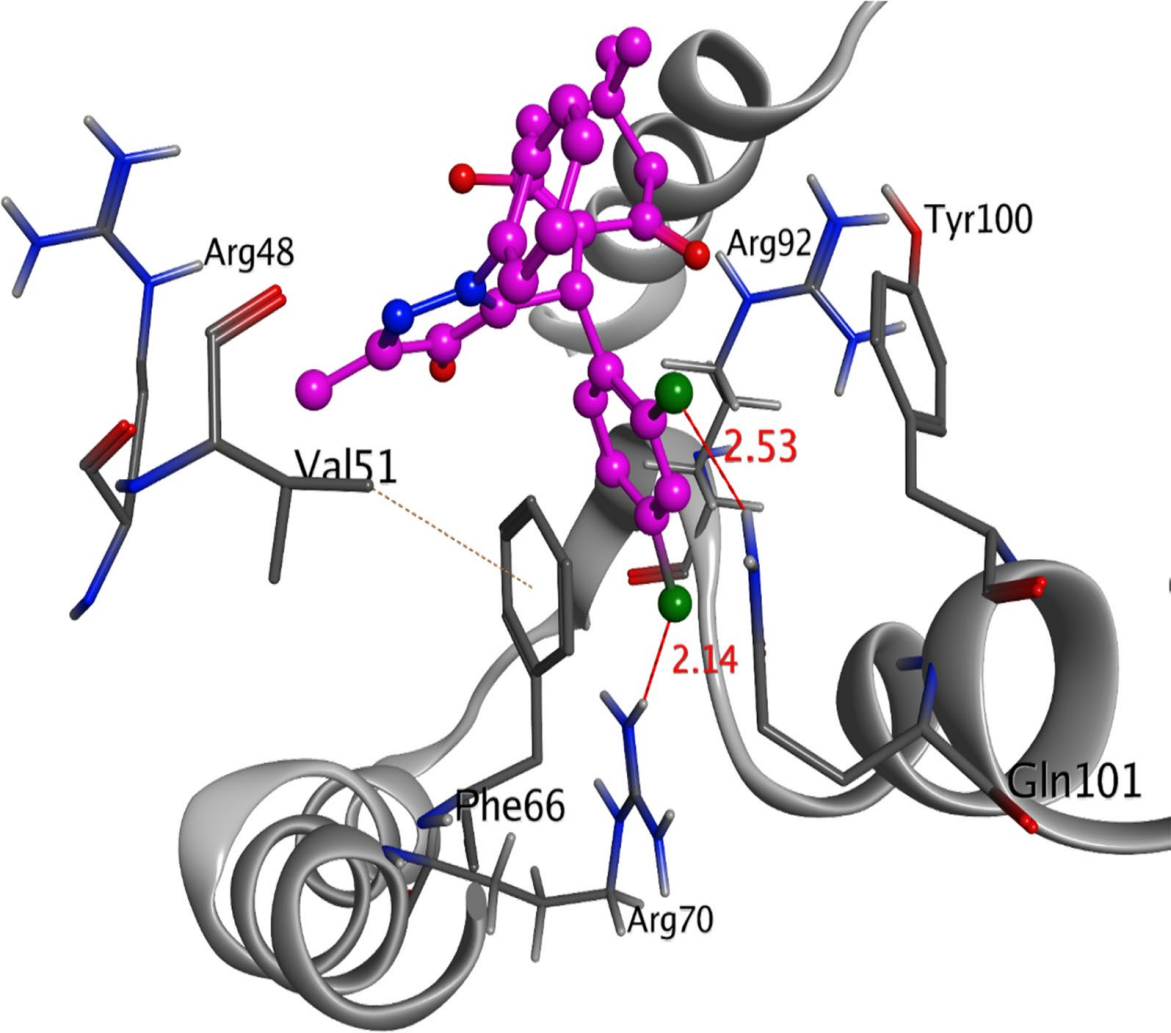
3-D interaction diagram for the compound **4l** (magenta) presenting a number of electrostatic (red dotted lines) and hydrophobic interactions (orange) with crucial residues of Thymidylate Kinase target protein (gray) from *S.aureus*

Among all derivatives, compound **4l** displayed the same electrostatic and hydrophobic interactions with crucial residues of TMK protein from *S. aureus*as presented by co-crystallized ligand. As illustrated in Fig. 2, the substituted part of compound **4l** moved inside the cavity where both chlorine atoms at 2 and 4 positions were engaged in the formation of two halogen bonds with the amino groups of Arg70 and Gln101 at 2.14 Å and 2.53 Å, respectively. Moreover, dichloro substituted benzene ring along with the pyrazole ring displayed various π–π and π-cation interactions with the crucial residues Phe66 and Arg92 of the target protein. Apart from it, the carbon atom located at R position and methyl of pyrazole ring were observed to establish hydrophobic interactions with Arg48 and Phe66 of TMK protein that might be responsible for their potent antibacterial activity.

**Fig. 3:**
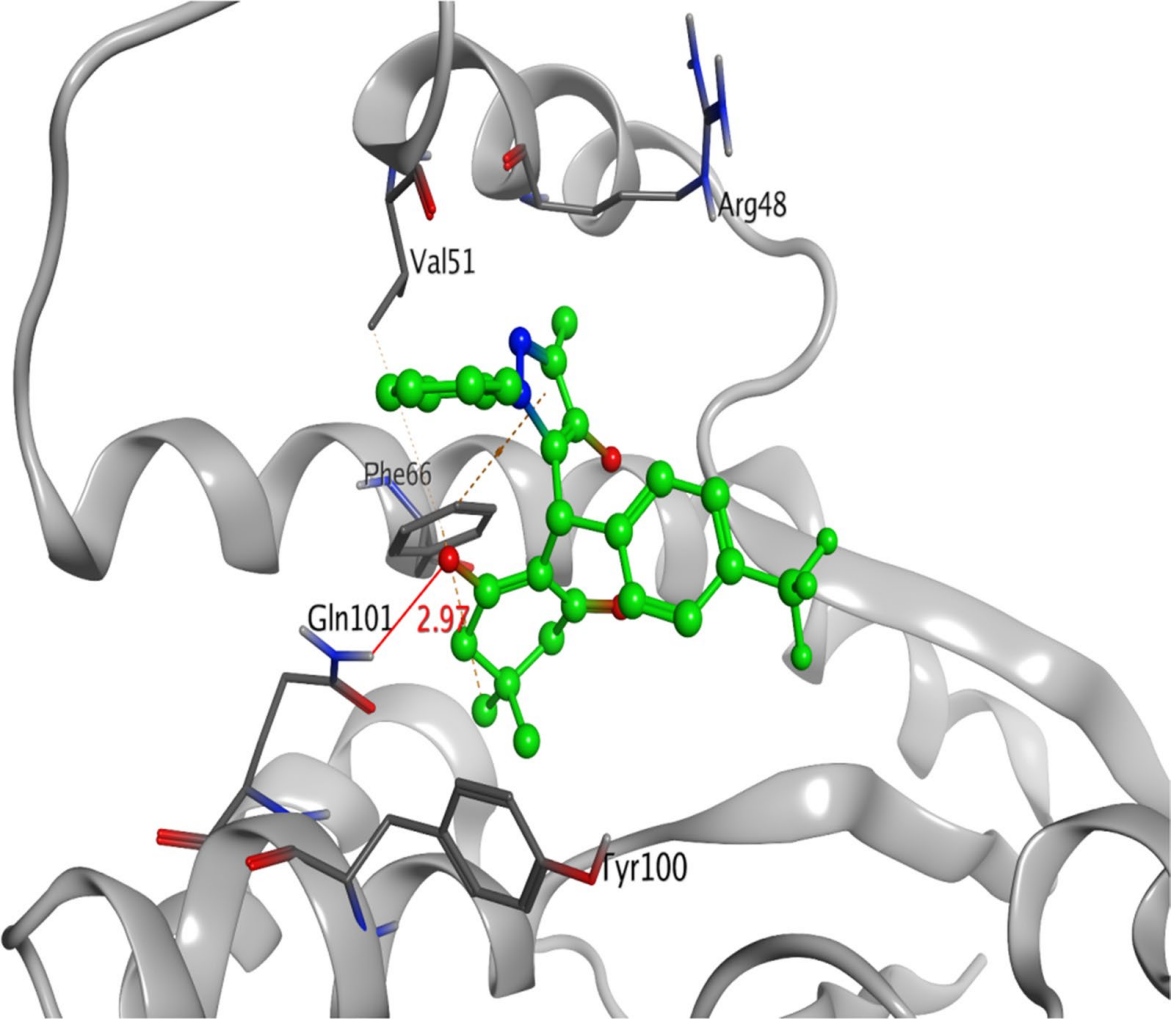
3D ribbon diagram of the active site of Thymidylate Kinase (grey) from *S. aureus* species displaying few electrostatic (red line) and multiple hydrophobic and π–π interactions with hotspot residues (hot pink) responsible for the moderate inhibitory activity of most potent compound **4k**

Comparatively, compound **4k** being the most active against *B. subtilis* species showed less or very few interactions with the TMK protein (4QGG) from *S. aureus* origin (Fig. 3).

**Fig. 4:**
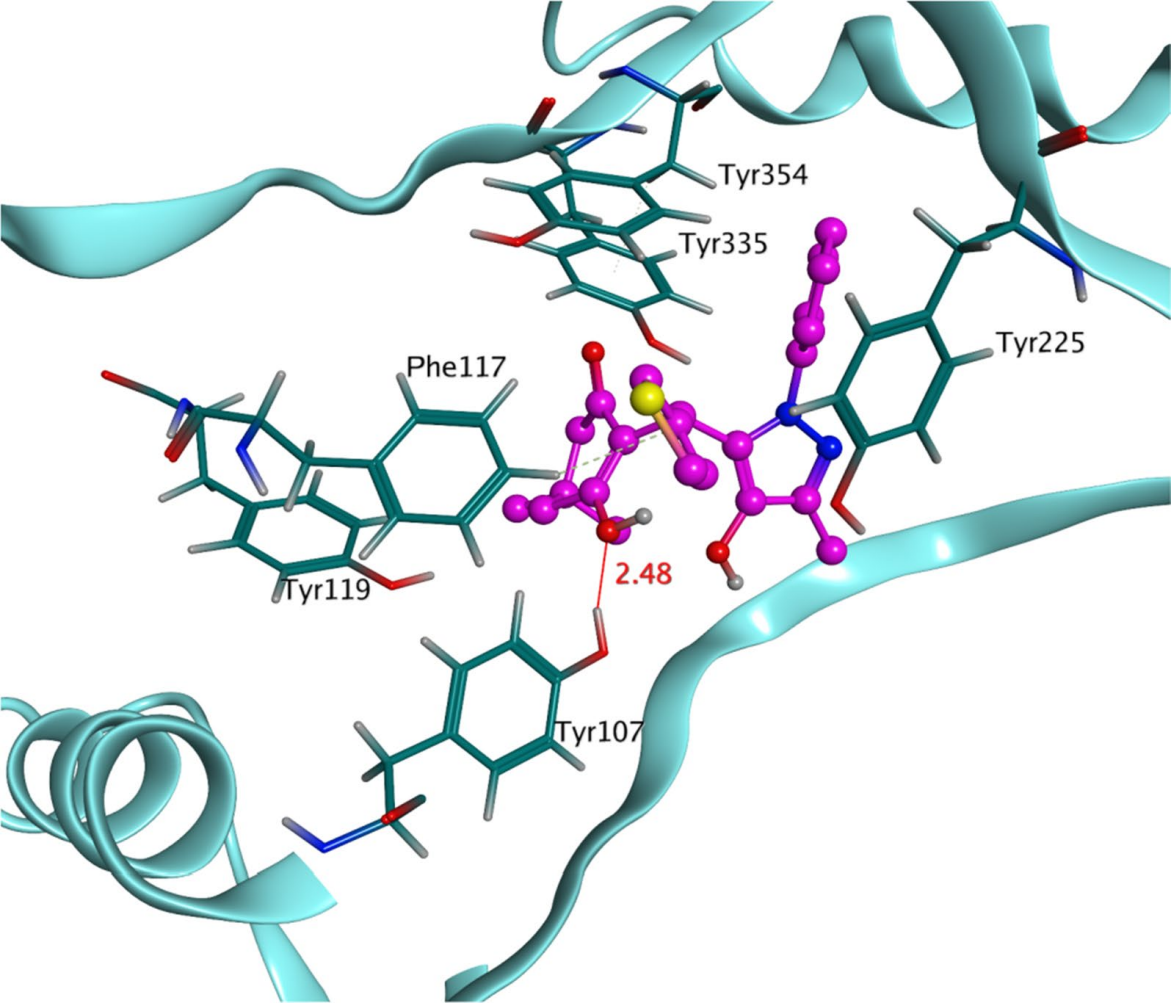
The post docking interaction map of most potent antifungal compound **4o** (magenta) exhibiting multiple types of interactions involving hydrophobic, π–π and electrostatic interactions (red lines) with the significant residues of antifungal target protein *N*-myristoyl transferase enzyme (light blue) from *C. albicans*

Similarly, the molecular visualization of **4o** revealed a number of significant electrostatic and hydrophobic interactions with the crucial residues of NMT. Figure 4 showed that the hydroxyl moiety attached at dimedone ring presented visible hydrogen bond with Tyr107 at a distance of 2.48 Å. Apart from it, three π–π interactions were observed among phenyl and thiol and hotspot residues Phe117, Tyr225 and Tyr 354. Simultaneously, several hydrophobic interactions were also noticed among compound **4o** and the crucial residues i.e. Tyr107, Phe 117, Tyr119, Tyr225, Tyr335. These results predicted TMK (*S. aureus*) and NMT (*C. albicans*) as the most probable targets for the antibacterial and antifungal activity of these newly synthesized agents.

## Conclusions

By using one-pot green protocol a series of pyrazole-dimedone derivatives **(4a–o)** were synthesized in high yields with a broad substrate scope under mild reaction conditions in water mediated by NHEt_2_. The requisite compounds were evaluated for their antibacterial and antifungal activities. After experimental investigations, structure–activity relationship profiling was predicted by ligand-based pharmacophore modeling highlighting three features as a requirement for their antimicrobial activity. While Molecular docking predicted the molecular mechanisms of these derivatives with seven different target proteins. Among them, TMK from *S. aureus* and NMT protein from *C. albicans* were predicted as the most suitable targets for the antibacterial and antifungal activities of these newly synthesized derivatives.

## Experimental

### Materials and methods

#### General

“All the chemicals were purchased from Aldrich, Sigma-Aldrich, Fluka etc., and were used without further purification, unless otherwise stated. All melting points were measured on a Gallenkamp melting point apparatus in open glass capillaries and are uncorrected. IR Spectra were measured as KBr pellets on a Nicolet 6700 FT-IR spectrophotometer. The NMR spectra were recorded on a Varian Mercury Jeol-400 NMR spectrometer. ^1^H-NMR (400 MHz), and ^13^C-NMR (100 MHz) were run in either deuterated dimethyl sulphoxide (DMSO-*d*_6_) or deuterated chloroform (CDCl_3_). Chemical shifts (*δ*) are referred in terms of *ppm* and *J*-coupling constants are given in Hz. Mass spectra were recorded on a Jeol of JMS-600 H. Elemental analysis was carried out on Elmer 2400 Elemental Analyzer; CHN mode”.

##### General procedure for Knoevenagel condensation Michael addition for the synthesis of **4a–o** (GP1)

A mixture of aldehyde **1** (1.5 mmol), 5,5-dimethylcyclohexane-1,3-dione **2,** (1.5 mmol), 3-methyl-1-phenyl-1*H*-pyrazol-5(4*H*)-one (1.5 mmol) and Et_2_NH (1.5 mmol, 155 μL) in 3 mL of degassed H_2_O was stirred at room temperature for 1–12 h until TLC showed complete disappearance of the reactants. The precipitate was removed by filtration and washed with ether (3 × 20 mL). Solid was dried to afford pure products **4a–o**.

##### *5*-*((2,4*-*Dichlorophenyl)(2*-*hydroxy*-*4,4*-*dimethyl*-*6*-*oxocyclohex*-*1*-*en*-*1*-*yl)methyl)*-*3*-*methyl*-*1*-*phenyl*-*1H*-*pyrazol*-*4*-*olate diethylaminium salt***4a**

**4a** was prepared according to the general procedure (**GP1**) from 2,4-dichlorobenzaldehyde yielding orange powdered materials. m.p: 144 °C; IR (CsI, cm^−*1*^): 3451, 2984, 2868, 2719, 2492, 1598, 1501, 1468, 1380, 1262; ^1^H-NMR (400 MHz, DMSO-*d*_6_): 8.08 (d, 1H, *J *= 7.3 Hz, Ph), 7.93 (d, H, *J* = 7.3 Hz, Ph), 7.42 (s, 1H, Ph), 7.32–7.04 (m, 5H, Ph), 4.96 (s, 1H, CH = C), 2.85 (q, 4H, *J* = 7.3 Hz, C*H*_2_CH_3_), 2.12 (s, 3H, CH_3_), 1.11 (t, 6H, *J* = 7.3 Hz, CH_2_C*H*_3_); ^13^C-NMR (100 MHz, DMSO-*d*_6_): *δ* = 157.6, 145.5, 142.4, 140.6, 132.1, 131.9, 128.3, 128.0, 126.6, 123.0, 119.1, 100.9, 41.7, 30.9, 13.2, 11.0; LC/MS (ESI): 330.07 [M]^+^for C_18_H_16_Cl_2_N_2_; Anal. for C_21_H_24_Cl_2_N_3_O; calcd C, 62.23; H, 5.97; Cl, 17.49; N, 10.37; Found: C, 62.23; H, 5.97; Cl, 17.49; N, 10.37.

##### *3*-*Hydroxy*-*2*-*((5*-*hydroxy*-*3*-*methyl*-*1*-*phenyl*-*1H*-*pyrazol*-*4*-*yl)(phenyl)methyl)*-*5,5*-*dimethylcyclohex*-*2*-*enone diethylaminium salt***4b**

**4b** was prepared according to the general procedure (**GP1**) from benzaldehyde yielding orange powdered materials. m.p: 102 °C; IR (CsI cm^−1^): 3448, 3058, 2957, 2732, 2507, 1582, 1579, 1501, 1492, 1454, 1365, 1263; ^1^H-NMR (400 MHz, DMSO-*d*_6_): *δ* 15.30 (s, 1H, OH), 7.92(m, 3H, Ph), 7.33–7.07 (m, 7H, Ph), 5.75 (s, 1H, benzyl-H), 2.86 (q, 4H, *J* = 7.3 Hz, C*H*_2_CH_3_), 2.16 (s, 3H, CH_3_), 2.12 (s, 2H, CH_2_), 2.09 (s, 2H, CH_2_), 1.11 (t, 6H, *J* = 7.3 Hz, CH_2_C*H*_3_), 1.10 (s, 3H, CH_3_), 1.00 (s, 3H, CH_3_); ^13^C-NMR (100 MHz, DMSO-*d*_6_): *δ* = 189.8, 157.2, 146.4, 145.8, 145.5, 140.5, 128.4, 128.3, 127.7, 127.2, 119.1, 102.2, 79.2, 41.4, 30.2, 28.8, 12.9, 12.7, 11.00; LC/MS (ESI): 262.1M]^+^ for C_18_H_18_N_2_; Anal. for C_29_H_38_N_3_O_3_; calcdC, 73.08; H, 8.04; N, 8.82; Found: C, 73.07; H, 8.05; N, 8.83.

##### *Diethylammonium 5-((4-chlorophenyl)(2-hydroxy-4,4-dimethyl-6-oxocyclohex-1-en-1-yl)methyl)-3-methyl-1-phenyl-1H-pyrazol -4-olate***4c**

**4c** was prepared according to the general procedure (**GP1**) from 4-chlorobenzaldehyde yielding orange powdered materials. m.p: 92 °C; IR (CsI cm^−1^): 3450, 2958, 2868, 2732, 2506, 1702, 1579, 1501, 1487, 1387, 1366, 1318, 1263; ^1^H-NMR (400 MHz, DMSO-*d*_6_): *δ* 15.30 (s, 1H, OH), 7.34–7.07 (m, 7H, Ph), 5.57 (s, 1H, benzyl-H), 2.91(q, 4H, *J* = 7.3 Hz, C*H*_2_CH_3_), 2.19 (s, 3H, CH_3_), 2.18 (s, 2H, CH_2_), 2.12 (s, 2H, CH_2_), 0.99(t, 6H, *J* = 7.3 Hz, CH_2_C*H*_3_), 1.14 (s, 3H, CH_3_), 1.15 (s, 3H, CH_3_); ^13^C-NMR (100 MHz, DMSO-*d*_6_): *δ* = 189.8, 157.2, 146.4, 145.8, 145.5, 140.5, 128.4, 128.3, 127.7, 127.2, 119.1, 102.2, 79.2, 41.4, 30.2, 28.8, 12.9, 12.7, 11.00; LC/MS (ESI): 262.1 M]^+^ for C_18_H_17_ClN_2_; Anal. for C_29_H_36_ClN_3_O_3_; Calcd C, 73.08; H, 8.04; N, 8.82; Found: C, 73.07; H, 8.05; N, 8.83, Cl, 6.21.

##### *3*-*Hydroxy*-*2*-*((5*-*hydroxy*-*3*-*methyl*-*1*-*phenyl*-*1H*-*pyrazol*-*4*-*yl)(p*-*tolyl)methyl)*-*5,5*-*dimethylcyclohex*-*2*-*enone diethylaminium salt***4d**

**4d** was prepared according to the general procedure (**GP1**) from *p*-tolualdehyde yielding orange powdered materials. m.p: 104 °C; IR (CsI, cm^−1^): 3450, 3017, 2956, 2732, 2506, 1683, 1581, 1501, 1455, 1386, 1318, 1260; ^1^H-NMR (400 MHz, CDCl_3_): *δ* 15.45 (s, 1H, OH), 7.67 (dd, 2H, *J* = 7.3 Hz, 1.5 Hz, Ph), 7.28 (dd, 2H, *J* = 7.3 Hz, 1.5 Hz, Ph), 7.20–6.94 (m, 5H, Ph), 5.62 (s, 1H, benzyl-H), 2.31 (s, 3H, CH_3_), 2.29 (s, 2H, CH_2_), 2.28 (s, 3H, CH_3_), 2.23 (s, 2H, CH_2_), 2.18 (q, 4H, *J* = 7.3 Hz, C*H*_2_CH_3_), 1.01 (s, 6H, CH_3_), 0.84 (t, 6H, *J* = 7.3 Hz, CH_2_C*H*_3_); ^13^C-NMR (100 MHz, CDCl_3_): *δ* = 189.8, 168.5, 157.9, 145.9, 140.4, 128.8, 128.7, 128.5, 127.6, 127.3, 121.7, 121.3, 80.3, 41.7, 31.5, 20.9, 12.6, 11.5; LC/MS (ESI): 276.1 [M]^+^ for C_19_H_20_N_2_; Anal. for C_30_H_40_N_3_O_3_; calcdC, 73.44; H, 8.22; N, 8.56; Found: C, 73.43; H, 8.23; N, 8.57.

##### *3*-*Hydroxy*-*2*-*((5*-*hydroxy*-*3*-*methyl*-*1*-*phenyl*-*1H*-*pyrazol*-*4*-*yl)(m*-*tolyl)methyl)*-*5,5*-*dimethylcyclohex*-*2*-*enone diethylaminium salt***4e**

**4e** was prepared according to the general procedure (**GP1**) from *m*-tolualdehyde yielding orange powdered materials. m.p: 97 °C; IR (CsI, cm^−1^): 3449, 3033, 2956, 2731, 2506, 1581, 1501, 1387, 1318, 1261; ^1^H-NMR (400 MHz, DMSO-*d*_6_): *δ* 15.45 (s, 1H, OH), 7.68 (dd, 2H, *J* = 7.3 Hz, 1.5 Hz, Ph), 7.63 (dd, 2H, *J* = 7.3 Hz, 1.5 Hz, Ph), 7.28–7.06 (m, 5H, Ph), 5.62 (s, 1H, benzyl-H), 2.30 (s, 3H, CH_3_), 2.20 (s, 2H, CH_2_), 2.23 (s, 3H, CH_3_), 2.18 (s, 2H, CH_2_), 2.25 (q, 4H, *J* = 7.3 Hz, C*H*_2_CH_3_), 1.00 (s, 6H, CH_3_), 0.83 (t, 6H, *J* = 7.3 Hz, CH_2_C*H*_3_); ^13^C-NMR (100 MHz, DMSO-*d*_6_): *δ* = 189.8, 168.5, 157.9, 145.9, 140.4, 128.8, 128.7, 128.5, 127.6, 127.3, 121.7, 121.3, 80.3, 41.7, 31.5, 20.9, 12.6, 11.5; Anal. for C_30_H_40_N_3_O_3_; calcdC, 73.44; H, 8.22; N, 8.56; Found: C, 73.43; H, 8.23; N, 8.57.

##### *2*-*((4*-*Bromophenyl)(5*-*hydroxy*-*3*-*methyl*-*1*-*phenyl*-*1H*-*pyrazol*-*4*-*yl)methyl)*-*3*-*hydroxy*-*5,5*-*dimethylcyclohex* -*2*-*enone diethylaminium salt***4f**

**4f** was prepared according to the general procedure (**GP1**) from *p*-bromobenzaldehyde yielding orange powdered materials. m.p: 86 °C; IR (KBr, cm^−1^): 3449, 2957, 2868, 2731, 250, 1699, 1579, 1501, 1483, 1388, 1263; ^1^H-NMR (400 MHz, DMSO-*d*_6_): *δ* 15.45 (s, 1H, OH), 7.91 (dd, 2H, *J* = 7.3 Hz, 1.5 Hz, Ph), 7.35–7.26 (m, 5H, Ph), 7.20–6.96 (dd, 2H, J = 7.3 Hz, 1.5 Hz, Ph), 5.50 (s, 1H, benzyl-H), 2.90 (q, 4H, *J* = 7.3 Hz, C*H*_2_CH_3_), 2.13 (s, 3H, CH_3_), 2.07 (s, 2H, CH_2_), 2.05 (s, 2H, CH_2_), 1.14 (t, 6H, *J* = 7.3 Hz, CH_2_C*H*_3_), 1.12 (s, 3H, CH_3_), 0.96 (s, 3H, CH_3_); ^13^C-NMR (100 MHz, DMSO-*d*_6_): *δ* = 189.8, 157.2, 155.9, 147.0, 145.8, 145.5, 140.7, 130.4, 129.6, 129.5, 128.4, 128.2, 122.9, 119.0, 118.8, 101.7, 79.7, 41.4, 31.9, 30.1, 28.3, 12.9, 128, 11.0; LC/MS (ESI): 340.1 [M]^+^ for C_18_H_17_BrN_2_; Anal. for C_29_H_37_BrN_3_O_3_; calcd C, 62.70; H, 6.71; Br, 14.38; N, 7.56; Found: C, 62.71; H, 6.71; Br, 14.39; N, 7.54.

##### *2*-*((3*-*Bromophenyl)(5*-*hydroxy*-*3*-*methyl*-*1*-*phenyl*-*1H*-*pyrazol*-*4*-*yl)methyl)*-*3*-*hydroxy*-*5,5*-*dimethylcyclohex* -*2*-*enone diethylaminium salt***4g**

**4g** was prepared according to the general procedure (**GP1**) from *m*-bromobenzaldehyde yielding rose powdered materials. m.p: 97 °C; IR (KBr, cm^−1^): 3447, 2957, 2868, 2730, 2505, 1584, 1501, 1470, 1388, 1365, 1262; ^1^H-NMR (400 MHz, DMSO-*d*_6_): *δ* 15.45 (s, 1H, OH), 7.92 (dd, 1H, *J* = 7.3 Hz, 1.5 Hz, Ph), 7.50 (s, 1H, Ph), 7.35–7.04 (m, 8H, Ph), 5.55 (s, 1H, benzyl-H), 2.89 (q, 4H, *J* = 7.3 Hz, C*H*_2_CH_3_), 2.15 (s, 3H, CH_3_), 2.09 (s, 2H, CH_2_), 2.06 (s, 2H, CH_2_), 1.14 (t, 6H, *J* = 7.3 Hz, CH_2_C*H*_3_), 1.10 (s, 3H, CH_3_), 0.98 (s, 3H, CH_3_); ^13^C-NMR (100 MHz, DMSO-*d*_6_): *δ* = 189.8, 157.2, 155.9, 149.3, 147.0, 145.8, 145.5, 140.7, 140.2, 129.9, 128.4, 128.3, 123.0, 119.0, 118.8, 101.6, 79.1, 41.4, 31.9, 30.1, 28.3, 12.9, 128, 11.0; LC/MS (ESI): 340.1 [M]^+^ for C_18_H_17_BrN_2_; Anal. for C_29_H_37_BrN_3_O_3_; calcd C, 62.70; H, 6.71; Br, 14.38; N, 7.56; Found: C, 62.71; H, 6.71; Br, 14.39; N, 7.53.

##### *3*-*Hydroxy*-*2*-*((5*-*hydroxy*-*3*-*methyl*-*1*-*phenyl*-*1H*-*pyrazol*-*4*-*yl)(4*-*nitrophenyl)methyl)*-*5,5*-*dimethylcyclohex*-*2*-*enone diethylaminium salt***4h**

**4h** was prepared according to the general procedure (**GP1**) from *p*-nitrobenzaldehyde yielding paige powdered materials. m.p: 106 °C; IR (CsI, cm^−1^): 3451, 2958, 2869, 2732, 2503, 1707, 1597, 1513, 1387, 1320, 1267; ^1^H-NMR (400 MHz, CDCl_3_): *δ* 15.40 (s, 1H, OH), 8.02 (dd, 2H, *J* = 7.3 Hz, 1.5 Hz, Ph), 7.61 (dd, 2H, *J *= 7.3 Hz, 1.5 Hz, Ph), 7.31–7.19 (m, 5H, Ph), 5.72 (s, 1H, benzyl-H), 2.70 (q, 4H, *J* = 7.3 Hz, C*H*_2_CH_3_), 2.27 (s, 3H, CH_3_), 2.24 (s, 2H, CH_2_), 2.19 (s, 2H, CH_2_), 1.07 (s, 6H, CH_3_), 1.02 (t, 6H, *J* = 7.3 Hz, CH_2_C*H*_3_); ^13^C-NMR (100 MHz, CDCl_3_): *δ* = 189.8, 157.9, 145.9, 140.4, 128.7, 128.6, 128.2, 127.9, 127.7, 125.3, 124.8, 121.6, 121.2, 80.3, 42.3, 31.6, 21.7, 11.4; LC/MS (ESI): 307.1 [M]^+^ for C_18_H_17_N_3_O_2_; Anal. for C_29_H_37_N_4_O_5_; calcd C, 66.77; H, 7.15; N, 10.74; Found: C, 66.75; H, 7.16; N, 10.75.

##### *3*-*Hydroxy*-*2*-*((5*-*hydroxy*-*3*-*methyl*-*1*-*phenyl*-*1H*-*pyrazol*-*4*-*yl)(3*-*nitrophenyl)methyl)*-*5,5*-*dimethylcyclohex2*-*enone diethylaminium salt***4i**

**4i** was prepared according to the general procedure (**GP1**) from *m*-nitrobenzaldehyde yielding white paige powdered materials. m.p: 99 °C; IR (CsI, cm^−1^): 3447, 3067, 2958, 2731, 2560, 1705, 1597, 1502, 1387, 1348, 1265; ^1^H-NMR (400 MHz, CDCl_3_): *δ* 15.30 (s, 1H, OH), 8.02(dd, 2H, *J* = 7.3 Hz, 1.5 Hz, Ph), 7.61 (dd, 2H, *J* = 7.3 Hz, 1.5 Hz, Ph), 7.31–7.19 (m, 5H, Ph), 5.72 (s, 1H, benzyl-H), 2.64 (q, 4H, *J* = 7.3 Hz, C*H*_2_CH_3_), 2.27 (s, 3H, CH_3_), 2.25 (s, 2H, CH_2_), 2.18 (s, 2H, CH_2_), 1.05 (s, 6H, CH_3_), 1.02 (t, 6H, *J* = 7.3 Hz, CH_2_C*H*_3_); ^13^C-NMR (100 MHz, CDCl_3_): *δ* = 189.8, 157.9, 145.9, 140.4, 128.7, 128.6, 128.2, 127.9, 127.7, 125.3, 124.8, 121.6, 121.2, 80.3, 42.3, 31.6, 21.7, 11.6; LC/MS (ESI): 307.1 [M]^+^ for C_18_H_17_N_3_O_2_; Anal. for C_29_H_37_N_4_O_5_; calcd C, 66.77; H, 7.15; N, 10.74; Found: C, 66.75; H, 7.16; N, 10.75.

##### *3*-*Hydroxy*-*2*-*((5*-*hydroxy*-*3*-*methyl*-*1*-*phenyl*-*1H*-*pyrazol*-*4*-*yl)(4*-*methoxyphenyl)methyl)*-*5,5*-*dimethylcyclo hex*-*2*-*enone diethylaminium salt***4j**

**4j** was prepared according to the general procedure (**GP1**) from anisaldehyde yielding deep orange materials. m.p: 84 °C; IR (CsI, cm^−1^): 3451, 2956, 2835, 2732, 2507, 1681, 1598, 1502, 1456, 1366, 1318, 1261; ^1^H-NMR (400 MHz, CDCl_3_): *δ* 15.35 (s, 1H, OH), 7.64 (dd, 2H, *J* = 7.3 Hz, 1.5 Hz, Ph), 7.27(dd, 2H, *J* = 7.3 Hz, 1.5 Hz, Ph), 7.14–6,68 (m, 5H, Ph), 5.59 (s, 1H, benzyl-H), 3.69 (s, 3H, OCH_3_), 2.33 (q, 4H, *J* = 7.3 Hz, C*H*_2_CH_3_), 2.27 (s, 3H, CH_3_), 2.25 (s, 2H, CH_2_), 2.17 (s, 2H, CH_2_), 0.99 (s, 6H, CH_3_), 0.83 (t, 6H, *J* = 7.3 Hz, CH_2_C*H*_3_); ^13^C-NMR (100 MHz, CDCl_3_): *δ* = 189.8, 157.9, 145.9, 140.4, 136.8, 128.8, 128.6, 125.4, 121.7, 121.3, 114.4, 113.4, 113.2, 80.3, 55.4, 41.7, 31.4, 11.2; LC/MS (ESI): 292.1 [M]^+^ for C_19_H_20_N_2_O; Anal. for C_30_H_40_N_3_O_4_; calcd C, 71.12; H, 7.96; N, 8.29; Found: C, 71.11; H, 7.97; N, 8.31.

##### *2*-*((4*-*Fluorophenyl)(5*-*hydroxy*-*3*-*methyl*-*1*-*phenyl*-*1H*-*pyrazol*-*4*-*yl)methyl)*-*3*-*hydroxy*-*5,5*-*dimethylcyclohex* -*2*-*enone diethylaminium salt***4k**

**4k** was prepared according to the general procedure (**GP1**) from *p*-fluorobenzaldehyde yielding orange powdered materials. m.p: 99 °C; IR (KBr, cm^−1^): 3450, 3.35, 2958, 2869, 2731, 2507, 1598, 1580, 1501, 1387, 1262; ^1^H-NMR (400 MHz, DMSO-*d*_6_): *δ* 15.45 (s, 1H, OH), 7.89–7.83 (dd, 2H, *J* = 7.3 Hz, 1.5 Hz, Ph), 7.32–7.28(dd, 2H, *J* = 7.3 Hz, 1.5 Hz, Ph), 7.20–6.94 (m, 5H, Ph), 5.53 (s, 1H, benzyl-H), 2.90 (q, 4H, *J* = 7.3 Hz, C*H*_2_CH_3_), 2.16 (s, 3H, CH_3_), 2.11 (s, 2H, CH_2_), 2.07 (s, 2H, CH_2_), 1.14 (t, 6H, *J* = 7.3 Hz, CH_2_C*H*_3_), 1.11 (s, 3H, CH_3_), 0.97 (s, 3H, CH_3_); ^13^C-NMR (100 MHz, DMSO-*d*_6_): *δ* = 189.8, 157.2, 147.0, 145.7, 140.2, 128.6, 128.5, 128.3, 123.3, 119.2, 118.9, 113.6, 102.4, 102.3, 79.2, 41.4, 31.3, 30.1, 28.7, 12.8, 12.6, 11.0; LC/MS (ESI): 280.1 [M]^+^ For C_18_H_17_FN_2_; Anal. for C_29_H_37_FN_3_O_3_; calcd C, 70.42; H, 7.54; F, 3.84; N, 8.50; Found: C, 70.43; H, 7.54; F, 3.83; N, 8.49.

##### *3*-*Hydroxy*-*2*-*((5*-*hydroxy*-*3*-*methyl*-*1*-*phenyl*-*1H*-*pyrazol*-*4*-*yl)(4*-*(trifluoromethyl)phenyl)methyl)*-*5, 5*-*dimethylcyclohex*-*2*-*enone diethylaminium salt***4l**

**4l** was prepared according to the general procedure (**GP1**) from *p*-trifluoromethylbenzaldehyde yielding yellow powdered materials. m.p: 96 °C; IR (CsI, cm^−1^): 3451, 2959, 2870, 2733, 2506, 1615, 1598, 1502, 1387, 1325, 1266; ^1^H-NMR (400 MHz, DMSO-*d*_6_): *δ* 16.45 (s, 1H, OH), 7.94–7.90 (dd, 2H, *J* = 7.3 Hz, 1.5 Hz, Ph), 7.57–7.44 (dd, 2H, *J* = 7.3 Hz, 1.5 Hz, Ph), 7.34–7.06 (m, 5H, Ph), 5.76 (s, 1H, benzyl-H), 2.91 (q, 4H, *J* = 7.3 Hz, C*H*_2_CH_3_), 2.19 (s, 3H, CH_3_), 2.12 (s, 2H, CH_2_), 2.10 (s, 2H, CH_2_), 1.15 (t, 6H, *J* = 7.3 Hz, CH_2_C*H*_3_),1.11 (s, 3H, CH_3_), 1.00 (s, 3H, CH_3_); ^13^C-NMR (100 MHz, DMSO-*d*_6_): *δ* = 157.2, 147.0, 145.7, 140.2, 128.6, 128.5, 128.3, 123.3, 119.2, 118.9, 113.6, 102.4, 102.3, 79.2, 41.4, 31.3, 30.1, 28.7, 12.8, 12.6, 11.0; LC/MS (ESI): 330.13 [M]^+^ for C_19_H_17_F_3_N_2_; Anal. for C_30_H_37_F_3_N_3_O_3_; calcd C, 66.16; H, 6.85; F, 10.46; N, 7.72; Found: C, 66.17; H, 6.86; F, 10.45; N, 7.71.

##### *5*-*((2,6*-*Dichlorophenyl)(2*-*hydroxy*-*4,4*-*dimethyl*-*6*-*oxocyclohex*-*1*-*en*-*1*-*yl)methyl)*-*3*-*methyl*-*1*-*phenyl*-*1H*-*py razol*-*4*-*olate diethylaminium salt***4m**

**4m** was prepared according to the general procedure (**GP1**) from 2,6-dicholorobenzaldehyde yielding deep orange powdered materials. m.p: 142 °C; IR (CsI, cm^−1^): 3459, 3117, 3061, 2973, 2834, 2479, 1657, 1646, 1596, 1500, 1431, 1311, 153; ^1^H-NMR (400 MHz, DMSO-*d*6): 8.08 (d, 1H, *J *= 7.3 Hz, Ph), 7.93 (d, H, *J *= 7.3 Hz, Ph), 7.42 (s, 1H, Ph), 7.32–7.04 (m, 5H, Ph), 4.96 (s, 1H, CH = C), 2.85 (q, 4H, *J* = 7.3 Hz, C*H*_2_CH_3_), 2.12 (s, 3H, CH_3_), 1.11 (t, 6H, *J* = 7.3 Hz, CH_2_C*H*_3_); ^13^C-NMR (100 MHz, DMSO-*d*_6_): *δ* = 161.6, 160.1, 150.0, 148.0, 132.9, 132.7, 131.3, 129.0, 128.9, 128.5, 128.1, 118.1, 117.8, 14.4; LC/MS (ESI): 330.07 [M]^+^for C_18_H_16_Cl_2_N_2_; Anal. for C_17_H_12_Cl_2_N_2_O; calcd C, 61.65; H, 3.65; Cl, 21.41; N, 8.46; Found: C, 61.64; H, 3.63; Cl, 21.40; N, 8.44.

##### *5*-*((2*-*Hydroxy*-*4,4*-*dimethyl*-*6*-*oxocyclohex*-*1*-*en*-*1*-*yl)(naphthalen*-*2*-*yl)methyl)*-*3*-*methyl*-*1*-*phenyl*-*1H*-*pyraz ol*-*4*-*olate diethylaminium salt***4n**

**4n** was prepared according to the general procedure (**GP1**) from naphthaldehyde yielding orange powdered materials. m.p: 102 °C; IR (CsI, cm^−1^): 3452, 3053, 2956, 2729, 2500, 1692, 1579, 1502, 1387, 1320, 1268; ^1^H-NMR (400 MHz, DMSO-*d*_6_): 15.32 (s, 1H, OH), 7.96–7.26 (m, 8H, Ph), 5.75 (s, 1H, benzyl-H), 2.27 (q, 4H, *J* = 7.3 Hz, C*H*_2_CH_3_), 2.20 (s, 3H, CH_3_), 2.01 (s, 2H, CH_2_), 2.00 (s, 2H, CH_2_), 1.06 (s, 6H, CH_3_), 0.64 (t, 6H, *J* = 7.3 Hz, CH_2_C*H*_3_);^13^C-NMR (100 MHz, DMSO-*d*_6_): *δ* = 192.3, 156.1, 146.7, 139.3, 128.7, 128.7, 126, 121.7, 121.30, 103.6, 78.8, 42.1, 31.3, 12.6; LC/MS (ESI): 312.0 [M]^+^ for C_22_H_20_N_2_; Anal. for C_27_H_36_N_3_O_3_S; calcd C, 67.19; H, 7.52; N, 8.71; S, 6.64; Found: C, 67.20; H, 7.52; N, 8.73.

##### *3*-*Hydroxy*-*2*-*((5*-*hydroxy*-*3*-*methyl*-*1*-*phenyl*-*1H*-*pyrazol*-*4*-*yl)(thiophen*-*2*-*yl)methyl)*-*5,5*-*dimethylcyclohex2*-*enone diethylaminium salt***4o**

**4o** was prepared according to the general procedure (**GP1**) from thiophenaldehyde yielding brown powdered materials. m.p: 87 °C; IR (KBr, cm^−1^): 3450, 3063, 2956, 2731, 2505, 1681, 1580, 1501, 1387, 1366, 1261; ^1^H-NMR (400 MHz, CDCl_3_): *δ* 15.32 (s, 1H, OH), 7.71–6.64 (m, 8H, Ph), 5.81 (s, 1H, benzyl-H), 2.47(q, 4H, *J* = 7.3 Hz, C*H*_2_CH_3_), 2.36 (s, 3H, CH3), 2.27(s, 2H, CH_2_), 2.23 (s, 2H, CH_2_), 1.12(s, 6H, CH_3_), 0.98(t, 6H, *J* = 7.3 Hz, CH_2_C*H*_3_); ^13^C-NMR (100 MHz, CDCl_3_): *δ* = 192.3, 156.1, 146.7, 139.3, 128.7, 128.7, 126, 121.7, 121.30, 103.6, 78.8, 42.1, 31.3, 12.6; LC/MS (ESI): 268.1 [M]^+^ for: C_16_H_16_N_2_S; Anal. for C_27_H_36_N_3_O_3_S; calcd C, 67.19; H, 7.52; N, 8.71; S, 6.64; Found: C, 67.20; H, 7.52; N, 8.73; S, 6.65.

### Antibacterial activity studies

The antimicrobial studies were carried out according to reported methodology in the following literature reported by Barakat et al. [12, 13, 23] including initial screening and determination of MIC.

### In-silico predictions

#### Pharmacophore modeling

A ligand-based pharmacophore model was developed by using MOE 2017 [24] suite. Where, a training set representing the most active lead analogs [12, 13] was selected, energy minimized and submitted to flexible alignment for analyzing the shared spatial arrangement of their pharmacophoric features. Generated hypotheses were ranked based on their accuracy scoring and atomic overlap. Among the highest ranked hypotheses, the best pharmacophore showing 100% accuracy was selected. This selected model was validated for its predictive efficacy by overlapping representative active analogs over it and calculating the RMSD (root mean square distance) between the query and mapped compounds.

#### Docking simulation

To predict the most suitable targets and inhibition mechanisms for the antibacterial and antifungal activities of the newly synthesized pyrazole-dimedone derivatives, reference compounds i.e. ciprofloxacin and fluconazole were submitted in Binding DB [25]. Binding DB works on the principle that similar compounds tend to have the same target proteins and seven proteins were chosen; four proteins i.e. Dihydrofolate Reductase (PDB ID 3FYV), Gyrase B (PDB ID 4URM), Thymidylate Kinase (TMK) (PDB ID 4QGG) and Sortase A (PDB ID 2MLM) from *S. aureus* for antibacterial (ciprofloxacin) and three proteins (Dihydrofolate Reductase (DHFR) (PDB ID 4HOF), Secreted Aspartic Protease (PDB ID 3Q70), and *N*-myristoyl transferase (PDB ID 1IYL) from *C. Albicans* for antifungal (fluconazole) compounds. The crystal structures of the seven target proteins were fetched from Protein Data Bank (www.rcsb.org/pdb) and all the proteins were prepared, charged, protonated and minimized via MOE 2016 suite. The chemical structures of synthesized compounds were built and saved in their 3D conformations by Builder tool incorporated in MOE 2016. Further protonation, minimization, charge application and atom-type corrections were also done by MOE 2016. Before docking, the efficiency of docking software was validated via redocking the crystallized ligand back into the pocket of significant antibacterial and antifungal target proteins. After redocking experiment (Additional file 1: Figures S1 and S2), we found MOE as the appropriate software to continue our in silico work with this software.

## Additional file

**Additional file 1.** Additional information.
