# Supplementary material for: Synthesis, antimicrobial activity, pharmacophore modeling and molecular docking studies of new pyrazole-dimedone hybrid architectures
Source: Chem Cent J. 2018 Mar 14;12:29. doi: 10.1186/s13065-018-0399-0 (PMC5852137; doi:10.1186/s13065-018-0399-0)
Supplement: Supplementary file 1 — Additional file 1. Additional information. [file 13065_2018_399_MOESM1_ESM.docx]

***SUPPORTING INFORMATION***

- 1. **Agar Cup plate method**

The tested bacterial strains were grown in Cation Adjustment Mueller-Hinton (CAMH) broth (Merck®, Darmstadt, Germany) while *C. albicans* strain was grown in Sabauraud Dextrose Broth (SDB) to mid-log phase. The bacterial and fungal suspension was measured spectrophotometery using Spectrophotometer (LKB® Ultrospec) at 625 nm to give absorbance 0.12 (1×10^8^ CFU/mL). The suspension was diluted 1:100 in CAMH broth to obtain 1×10^6^ CFU/mL. This suspension was swabbed on a CAMH agar plate (Merck®, Darmstadt, Germany) and allowed to dry completely. Mueller-Hinton Agar and Sabauraud Dextrose Agar were used for bacteria and fungi respectively. Four wells (7 mm in diameter) were made in agar plate using cork borer. A 1 mL of stock solution (5120 μg/mL) was 2-fold diluted in 1 mL DMSO to obtain 2560 μg/mL. A 100 µL (256 µg) of the tested compound was poured in the well using calibrated pipette. The plates were kept in refrigerator at 4 ^o^C for half an hour to allow diffusion of the compound in the agar. Then, the plates were incubated at 37 ^o^C for 24 h. After the incubation period, the diameter of the inhibition zone was measured and recorded in mm by aid of ruler. Ciprofloxacin (10 µg/cup) and fluconazole (10 µg/mL) were used as positive controls for antibacterial and antifungal respectively. The experiment was carried out in duplicate and the mean diameter was taken [1].

- 1. **Determination of MIC**

MIC was determined for the compounds that showed antimicrobial activity by cup plate method. Briefly, 2 mL of CAMH broth (for bacterial strains) and 2 mL of SAB (for fungal strain) was dispensed into 7 mL Peju sterile tubes. For each compound, 14 tubes were used. Tubes number 13 and 14 were used as positive growth control (No tested compound) and negative control for medium sterility (No microorganism) respectively. A 1 mL of stock solution (5120 μg/mL) was 10-fold diluted in 9 mL CAMH to obtain 512 μg/mL. A 2 mL of the tested compounds (512 μg/mL) was pipetted into the first tube and mixed well. Then 2 mL was withdrawn from 1^st^ tube and added to the 2^nd^ tube to make a twofold dilution. This procedure was repeated down to 12^th^ tube to reach the concentration of 0.125 μg/mL. A 2 mL was discarded from 12^th^ tube. A volume of 2 mL of inoculums (1×10^6^ CFU/mL) were added to all tubes except tube number 14 to give final 1×10^6^ CFU/mL. Ciprofloxacin and fluconazole were used as positive controls for antibacterial and antifungal, respectively. The inoculated tubes were incubated at 37 ^0^C for 20 h. After the incubation period, the results of MIC were recorded manually and interpreted according to the guidelines of British Society of Antimicrobial Chemotherapy (BSAC) [1].

## Methodology for the Molecular docking studies

To predict a specific target and mechanism of action for the antibacterial and antifungal activity of newly synthesized pyrazol-dimedone derivatives, seven different target proteins were predicted from Binding DB (https://www.bindingdb.org). Secreted Aspartic Protease (PDB ID 3Q70), Dihydrofolate Reductase (DHFR) (PDB ID 4HOF), and *N*-myristoyl Transferase (PDB ID 1IYL) from *C. Albicans* were selected as fungal target while for bacterial targets, four proteins involving Dihydrofolate Reductase (PDB ID 3FYV), Gyrase B (PDB ID 4URM), Thymidylate Kinase (TMK) (PDB ID 4QGG) and Sortase A (PDB ID 2MLM) from *S. aureus* were selected and fetched from Protein Data Bank ([www.rcsb.org/pdb](http://www.rcsb.org/pdb)) for this study. Among all these seven proteins, only two proteins i.e. one protein (Thymidylate Kinase) from *S. aureus* [2,3] and one protein (N-myristoyl Transferase) from *C. albican* [4] were preferred as they showed good binding affinities and molecular interactions with the synthesized compounds in docking simulation by MOE 2015 [5] while all other targets showed very few or no interactions with these derivatives.

Before docking, structures of synthesized compounds were built and saved in their 3D conformation by MOE 2015. Further protonation, minimization, charge application and atom-type corrections were also done by MOE 2015. Moreover, for target protein preparation, all the seven proteins were prepared, charged, protonated and minimized *via* the same software i.e. MOE 2015. Among all the crystal structures, the proteins presenting good binding affinity were preferred and selected to evaluate the activity of these compounds. 1IYL and 4QGG are the taregts for anti-fungal and antibacterial respectively. Chain A of all three proteins (4URM, 1IYL, and 4QGG) were selected for further studies. Re-docking were performed in order to evaluate the reliability of software to establish the correct binding pose. For docking, default MOE docking parameters i.e. Triangle Matcher Algorithm with two rescoring functions London dG and GBVI/WSA dG were utilized to generate 30 poses of each compound. As a result, the mdb output files were generated enclosing all docking results with scoring and multiple conformations of ligands. After docking, the best poses were analysed to figure out the most potent and effective antifungal and antibacterial inhibitor by visualizing the electrostatic and hydrophobic interactions of pyrazol-dimedone derivatives within binding pocket of above-mentioned proteins.

**
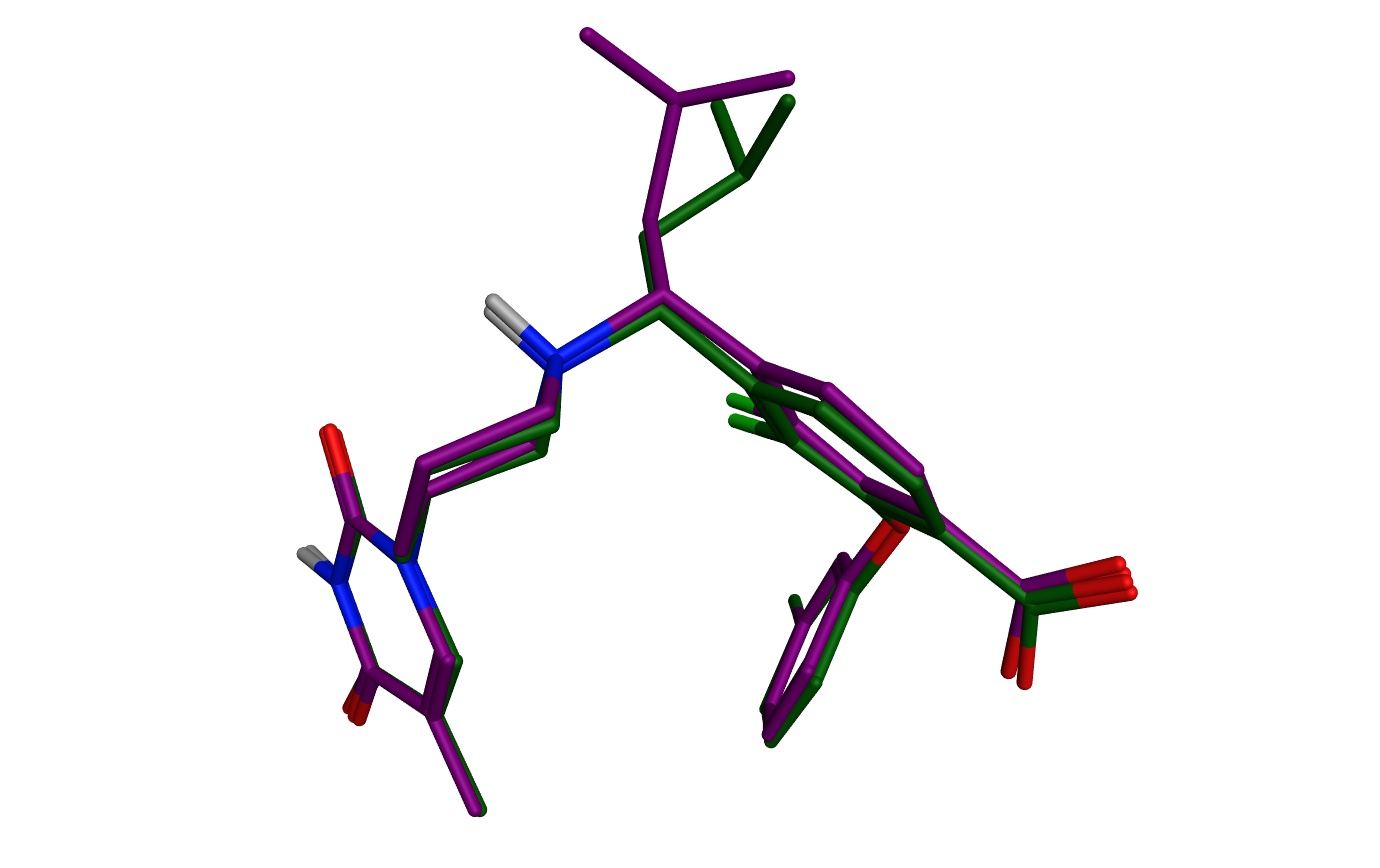
**

**Figure S1**: Re-docked pose of the Thymidylate kinase. Green color shows cognate ligand while magenta color shows the redocked pose

**
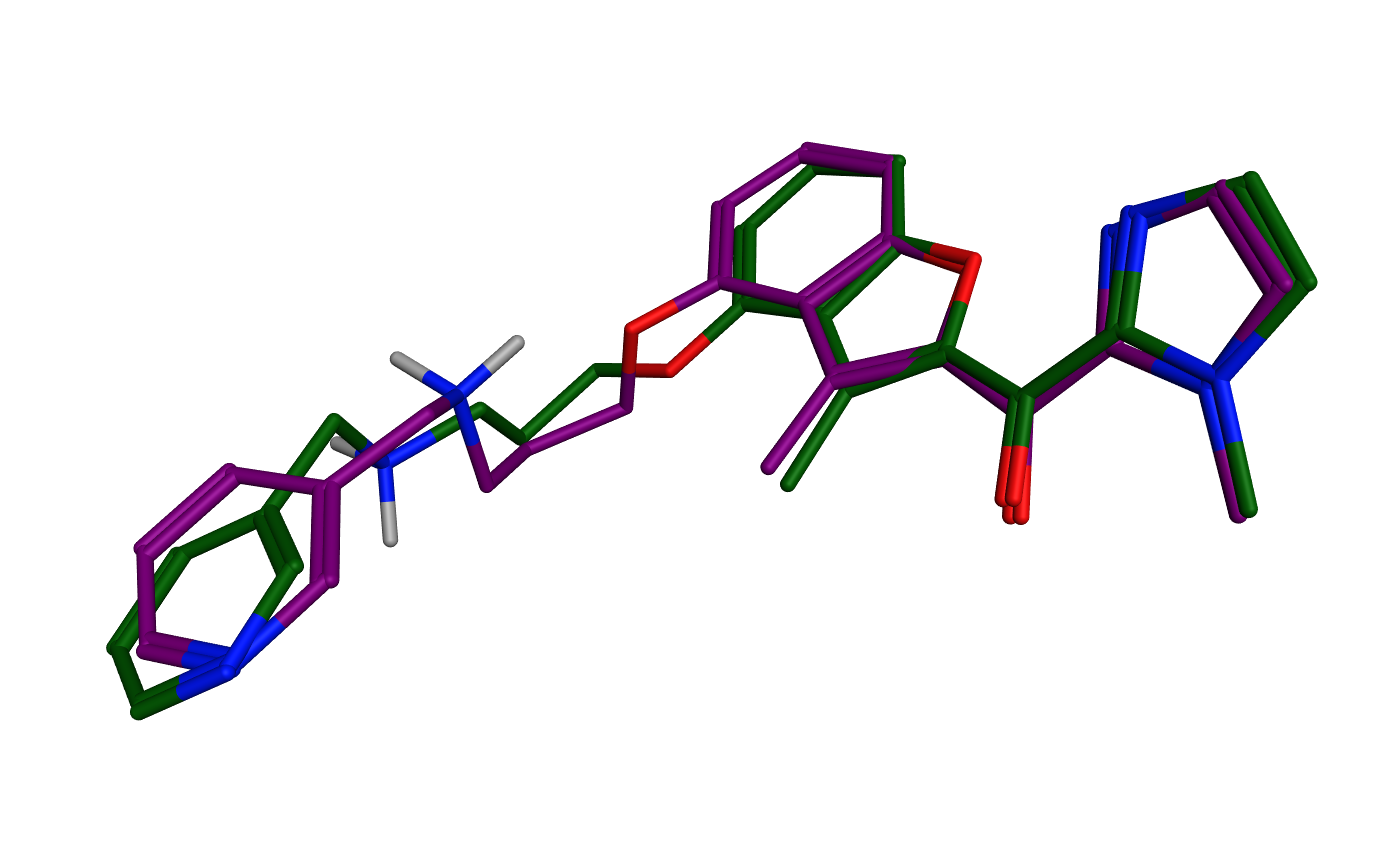
**

**Figure S2:** Re-docked pose of the *N*-myristoyl Transferase. Green color shows cognate ligand while magenta color shows the redocked pose

References

1. BSAC. Methods for Antimicrobial Susceptibility Testing—Version 14 Available at: <http://bsac.org.uk/wp-content/uploads/2012/02/BSAC-Susceptibility-testing-version-14.pdf>(Last accessed 9 April 2015).
2. Kawatkar SP, Keating TA, Olivier NB, Breen JN, Green OM, Guler SY, Hentemann MF, Loch JT, McKenzie AR, Newman JV (2014) Antibacterial inhibitors of gram-positive thymidylate kinase: Structure–activity relationships and chiral preference of a new hydrophobic binding region. J Med Chem 57:4584-4597.
3. Lu J, Patel S, Sharma N, Soisson SM, Kishii R, Takei M, Fukuda Y, Lumb KJ, Singh SB (2014) Structures of kibdelomycin bound to staphylococcus aureus GyrB and ParE showed a novel U-shaped binding mode. ACS chem. Biol. 9:2023-2031.
4. Sogabe S, Masubuchi M, Sakata K, Fukami TA, Morikami K, Shiratori Y, Ebiike H, Kawasaki K, Aoki Y, Shimma N (2002) Crystal structures of candida albicans N-myristoyltransferase with two distinct inhibitors. Chemistry & biology 9:1119-1128.
5. Molecular Operating Environment (MOE), 2013.08; Chemical Computing Group Inc., 1010 Sherbooke St. West, Suite #910, Montreal, QC, Canada, H3A 2R7, 2016.
